# Osteoimmune regulation underlies oral implant osseointegration and its perturbation

**DOI:** 10.3389/fimmu.2022.1056914

**Published:** 2023-01-24

**Authors:** T. Albrektsson, P. Tengvall, L. Amengual, P. Coli, G. A. Kotsakis, D. Cochran

**Affiliations:** ^1^ Department of Biomaterials, University of Gothenburg, Gothenburg, Sweden; ^2^ Dental Implantology Unit, Hospital Leonardo Guzmán, Antofagasta, Chile; ^3^ Edinburgh Dental Specialists, Edinburgh, United Kingdom; ^4^ Department of Prosthetic Dentistry and Dental Material Science, The Sahlgrenska Academy at Gothenburg University, Gothenburg, Sweden; ^5^ Department of Dental Material Science, The Sahlgrenska Academy at Gothenburg University, Gothenburg, Sweden; ^6^ Department of Periodontology, University of Texas, San Antonio, TX, United States

**Keywords:** bone healing, bone regeneration, osteoimmunology, immune reaction, osteomechanobiology, osteometabolics, osteoneurology, revascularization

## Abstract

In the field of biomaterials, an endosseous implant is now recognized as an osteoimmunomodulatory but not bioinert biomaterial. Scientific advances in bone cell biology and in immunology have revealed a close relationship between the bone and immune systems resulting in a field of science called osteoimmunology. These discoveries have allowed for a novel interpretation of osseointegration as representing an osteoimmune reaction rather than a classic bone healing response, in which the activation state of macrophages ((M1–M2 polarization) appears to play a critical role. Through this viewpoint, the immune system is responsible for isolating the implant biomaterial foreign body by forming bone around the oral implant effectively shielding off the implant from the host bone system, i.e. osseointegration becomes a continuous and dynamic host defense reaction. At the same time, this has led to the proposal of a new model of osseointegration, the foreign body equilibrium (FBE). In addition, as an oral wound, the soft tissues are involved with all their innate immune characteristics. When implant integration is viewed as an osteoimmune reaction, this has implications for how marginal bone is regulated. For example, while bacteria are constitutive components of the soft tissue sulcus, if the inflammatory front and immune reaction is at some distance from the marginal bone, an equilibrium is established. If however, this inflammation approaches the marginal bone, an immune osteoclastic reaction occurs and marginal bone is removed. A number of clinical scenarios can be envisioned whereby the osteoimmune equilibrium is disturbed and marginal bone loss occurs, such as complications of aseptic nature and the synergistic activation of pro-inflammatory pathways (implant/wear debris, DAMPs, and PAMPs). Understanding that an implant is a foreign body and that the host reacts osteoimmunologically to shield off the implant allows for a distinction to be drawn between osteoimmunological conditions and peri-implant bone loss. This review will examine dental implant placement as an osteoimmune reaction and its implications for marginal bone loss.

## 1 Introduction

Osseointegration is needed for oral implant function. Provided that properly trained individuals place clinically controlled oral implant systems, the general outcome is most positive with 10 year failure rates varying between 0-4% (1), and osseointegrated oral implants have in case studies been shown to function over 50 years in the body (2). However, the original view of osseointegration as just a simple bone repair process after osteotomy does not appear to be valid. As demonstrated originally by Donath and co-workers (3), an implant is recognized as a non-self material by the immune system of the body, i.e, in successfully osseointegrated cases. This was recently demonstrated through a quantitative polymerase chain reaction (qPCR)- and histological animal model study where the host established a clear and regulated inflammatory response which thereafter shielded-off the implanted biomaterial in bone (4). Therefore, what is seen when implants are placed in the hard tissues is an *Osteoimmune reaction*, a term that would better describe actual tissue reactions than the original term osseointegration. A recently published suggested definition reads “Osseointegration is a foreign body reaction where interfacial bone is formed as a defense reaction to shield off the implant from the tissues” (5).

**Figure 1:**
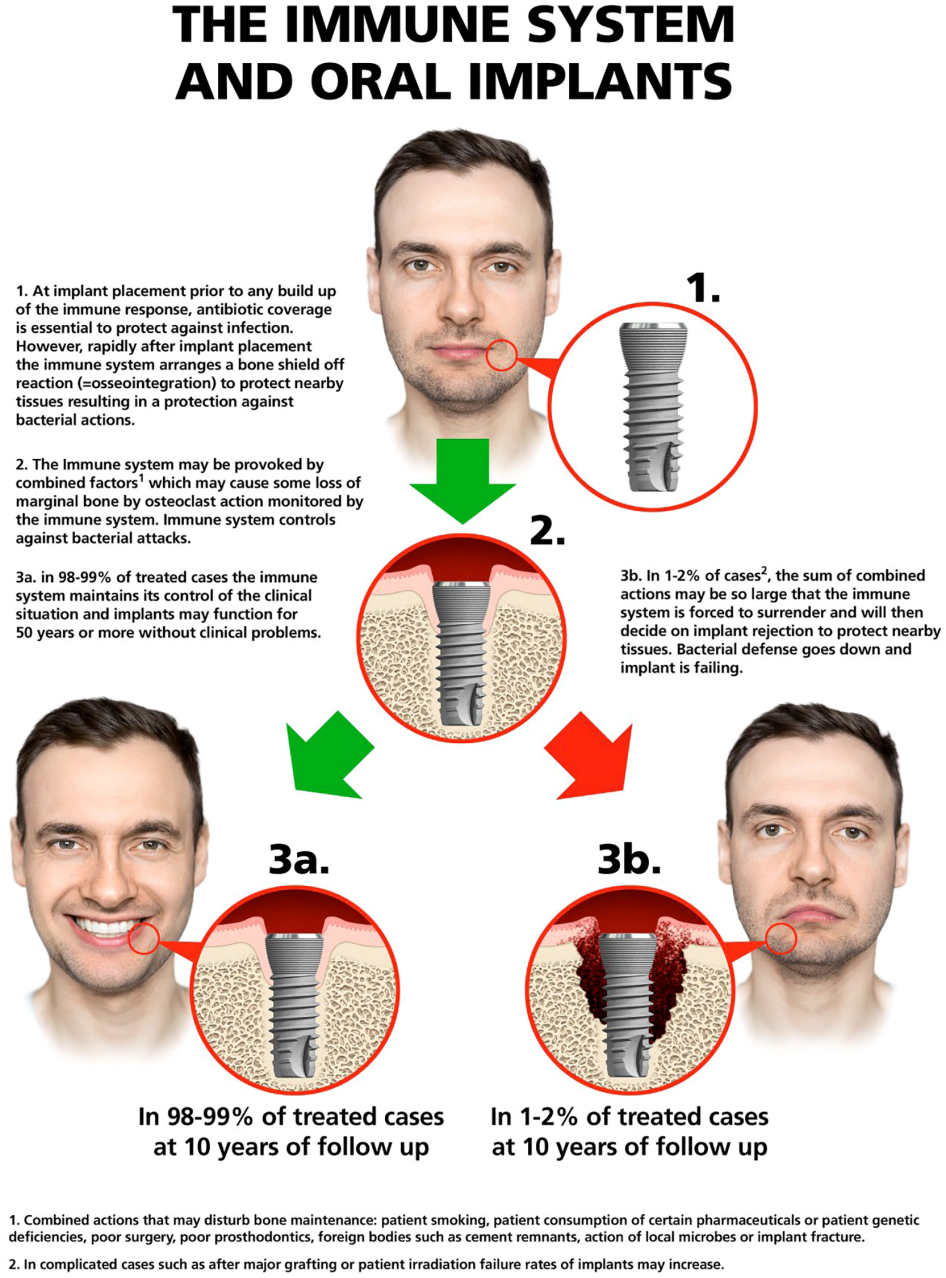
A general overview of immune system actions in relation to oral implants. Computerized image of human face.

In the vast majority of cases the immunological/inflammatory response mounted by the host will lead to implant integration rather than its rejection. Due to the immunologically and mechanically stimulated bone shield-off reaction and the osteoimmune/immunological equilibrium that is established in the case of oral implants, clinicians may load the implants that will then survive for many years in function. However, the immune and healing responses are not only transient one-time reactions, but instead represent a temporal continuum of dynamic hard and soft tissues changes (6). Therefore, today the focus is on modulation of the osteoimmune microenvironment at the bone-implant interface (7–9), understanding that if the host-biomaterial equilibrium becomes perturbed, the result can be marginal bone loss (MBL) or peripheral bone loss around the implant. If the temporal shift in equilibrium at the marginal bone is limited, MBL may be small and does not necessarily challenge the implant’s long term survival i.e a new host-biomaterial equilibrium is established (6). However, if continuous and of substantial magnitude, the provocation may result in a shift in the immune/re-balancing response from shielding off the implant to rejection of it (**Figure 1**). Taken together, these observations confirm differences between the teeth of an individual and implants – rules that apply to the former are generally irrelevant for the latter and vice versa. MBL around implants, in this context, should be considered a condition rather than a disease (10, 11). This paper aims to present an overview of osteoimmunology of relevance for osseointegration and threats to this condition, and to furthermore, analyze the situation from a bone cell/tissue point of view. We start with an overview of osteoimmunology and oral microbiology and discuss then perturbation of osteoimmune responses and marginal bone loss from different perspectives. The importance of the implant passivation layer is presented as well as potential sequale of primary and secondary corrosion phenomena. The paper ends with concluding remarks centered on the paradigm shift that is the result of a greater understanding of osteoimmunology, a core area of knowledge for interpreting implant outcome.

## 2 Basics of osteoimmunology

Traditionally, three types of bone cells have been described in bone tissue; osteoblasts, osteoclasts and osteocytes. Osteoblasts are responsible for bone growth and osteoclasts favor bone resorption. Activities of both depend on signaling cues (cytokines) and cell-cell interactions. Especially prominent is the receptor activator of nuclear factor κ B (RANK)-receptor activator of nuclear factor-kappa B ligand (RANKL) interaction. Osteocytes, which act in response to mechanical stimuli largely control the osteoclastic/osteoblastic activity through both net bone growth (via e.g. parathyroid hormone PTH, osteocalcin, mechanical stimuli and Wnt ligands) and bone resorption (via e.g. mechanical unloading, sclerostin and dickkopf signals (12)). The always ongoing bone remodeling process has thus traditionally been described as the carefully coordinated interaction between osteoblastic, osteocytic and osteoclastic activities, a process that is carried out by active basic multicellular units (BMUs) (13). Further, it has been described that the activity of RANKL and consequently osteoclastogenesis, is controlled *via* production of osteoprotegerin (OPG) by osteoblasts and other stromal cells. Hence, the OPG/RANKL balance has been proposed as the determining factor to maintain bone density (14).

In addition, a number of molecular and cellular mechanisms constitute a permanent interaction between bone tissue and the immune/inflammatory system. In this sense, the cells of both systems share common origins, since osteoclasts originate from stem cells of the monocyte-macrophage hematopoietic stem cell lineage and osteoblasts from the mesenchymal stem cell lineage (15). Furthermore, lymphocytic, dendritic cell and macrophage cytokines are all known to act as local bone remodeling regulatory factors (16). The molecular basis of the underlying mechanisms was identified only 20 years ago with the discovery of the essential role of the RANK/RANKL axis in bone and immune cell physiopathology. From that moment, the term “osteoimmunology” was coined to define a new discipline covering the interplay between bone and the immune systems (17).

A rapid evolution in our knowledge of immunology has taken place during the past decades. The adaptive immune response (mainly *via* T and B cells) was long thought to drive innate immunity. However, immunology had it backwards, as now macrophages and the innate immunity are increasingly in the focus of attention, not least in oral implantology. Indeed, because of the discovered macrophage polar-opposite kill and repair activities, the independence of these responses from T cells, and that these types of responses stimulate Th1- or Th2-type responses, macrophages were renamed M1 and M2 to highlight the importance of innate immunity (18). In recent years, it is also understood that bone formation and remodeling are influenced by the inflammatory state of the local microenvironment. In this regard, the eventual phenotypic switch of M1 to M2 macrophage seems to play a crucial role in modulating osteogenesis (19). Moreover, it has been proposed that an efficient and timely switch from M1 to M2 macrophage phenotype facilitates an osteogenic cytokine release and with it the formation of new bone tissue around implanted biomaterials. This is the basis for the concept of an osteoimmunomodulatory material (20). This was confirmed for titanium implants e.g. by Trindade´s works since 2018 (significantly up-regulated ARG1 gene expression around titanium at 10 days) (4, 21, 22). In relation to this, it has been postulated that mainly bone macrophages (osteomacs) would be responsible for the recruitment of osteoprogenitor cells to build new peri-implant bone, since the surface of the titanium implant would directly induce differentiation towards a pro-regenerative M2 macrophage (23). In addition, it is known that once macrophages acquire a functional polarization, they still retain the ability to continue changing in response to new environmental stimulation (24). This was shown in a recently detailed mapping of the mouse mandibular alveolar bone where a unique immune microenvironment was demonstrated under active bone remodeling and immunomodulation (25, 26).

**Figure 2:**
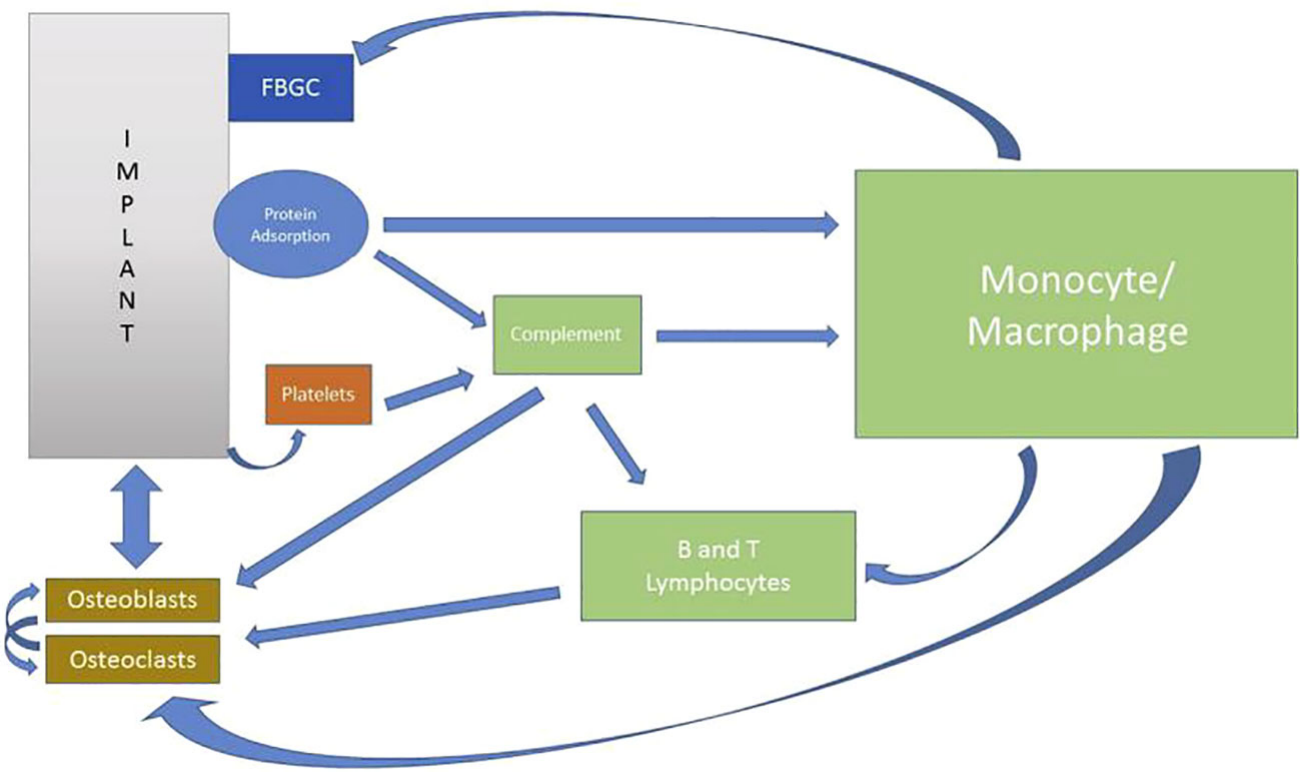
Hypothetical model for osseointegration dynamics. FBGC: foreign body giant cells. From Trindade, et al. Ref. (27).

All these findings indicate that oral osseointegration is maintained in a dynamic and likely immunologically dynamic environment. With this in mind, a new dynamic model of osseointegration has been proposed to represent an interplay between the complex osteoimmune/inflammatory events and oral implants, coined the Foreign Body Equilibrium (FBE). This model has in turn allowed a view of marginal bone loss (MBL) around oral implants to be a result of FBE susceptibility to peri-implant environmental conditions (27), (**Figure 2**). Therefore, MBL can be viewed as a biological, and maybe transient, imbalance in the local immune/inflammatory state (28) adjacent to artificial devices instead of as a disease (10).

## 3 Osseointegration and oral microbiology

The first study in which a direct bone anchorage to titanium was suggested as a clinical possibility was published in 1969 (29), and the term “osseointegration” was first coined in 1977 (30). After that and based on classical bone physiology and fracture healing studies, oral implants were considered bio-inert (31) and were considered similar to teeth by some investigators. Since teeth may suffer from periodontitis, it has been assumed that oral implants are also subjected to a hereditary inflammatory disease with relation to bacteria. Hence, the term peri-implantitis was introduced and seen as a bacterially related disease of oral implants (32). Over the last two decades this opinion about MBL has been accepted at several meetings arranged for the purpose of consensus (33). After these conferences, the discussion has continued “mirroring” the progression of gingivitis to periodontitis where peri-implant mucositis is assumed to precede peri-implantitis. However, features or conditions characterizing the conversion from peri-implant mucositis to peri-implantitis have not been identified, despite the scientific advances of the last decades (34). However, during the latest years large progress has been made in oral microbiology, with significance also to implants. For example, oral bacteria have the capability to produce mucosa and bone degrading peptides (35, 36), but are largely balanced by the presence and activities of B-cells, neutrophils, and different T-cells and their molecular products. In addition, the inherent immunomodulating role of the biomaterial and its interplay with the host’s innate immunity has been ignored. In fact, the fate of a bone implant appears to be largely determined by its effects on the host immune response. In general, persisting inflammation impedes tissue repair and favors bacterial overgrowth. Therefore, a balanced inflammatory environment around a biomaterial is critical, since both downregulated and excessive inflammatory responses lead to suboptimal bone regeneration clinically (37).

## 4 Perturbation of osteoimmune reactions

There appears to be two principal reasons for perturbation of the osteoimmune equilibrium in the area of the marginal bone around osseointegrated implants; septic and aseptic reactions:

### 4.1 Septic reactions

Currently, MBL is considered mostly to be due to septic reactions as evidence has emerged that bacteria can be present also in bone tissue itself. Apparently healed alveolar bone in the dental implant bed displayed bacterial species that further were found locally in the bone even in som cases of tooth agenesis (38, 39). The assumed mechanism of septic causes for MBL is bacterial recuitment of inflammatory bone resorbing cells (40) that may result in implant failure if the infection is maintained. The plethora of bacteria everywhere in the oral cavity may be interpreted as a substantial threat for implant survival. However, in reality oral implants fare quite well despite all bacteria. Analyzing situations where bacteria are known to cause clinical problems with implants include the case of oral implants placed without simultaneous antibiotic coverage, with a consequent increase in implant failure rates (41). In addition, bacteria can secondarily cause MBL (40) in the case of oral implants where a failing process has already been initiated for other reasons. It is of particular interest that these two situations with known possibilities for infection occur either prior to completed osseointegration or once the process of osseointegration failure has already begun. Considering the very high implant survival rates over long periods of time (42), such observations indicate the presence of very strong bacterial defense mechanisms as an inherent capacity of the body, and hence favor osseointegration. This bacterial defense was initially regarded synonymous with the establishment of hemi-desmosome formations (30). More recently, cellular mechanisms have been regarded as the reason for the defense such as a combination of inflammatory and immune cell types or keratinocytes (28, 43). Other potential mechanisms coupled to the defense may be associated with the immune reaction per se, a reaction inevitable in the case of oral implant placement. Another septic reaction close to implants may be seen originating from bacterial leakage between implant parts. However, this type of septic reaction is local and is not known to, on its own, generalize to attacks on the osseointegration process (26). In other words, presence of bacteria is inevitable in the oral cavity, but particular defense mechanisms may guard against bacterial actions in form of marginal bone resorption.

### 4.2 Aseptic reactions

**Figure 3:**
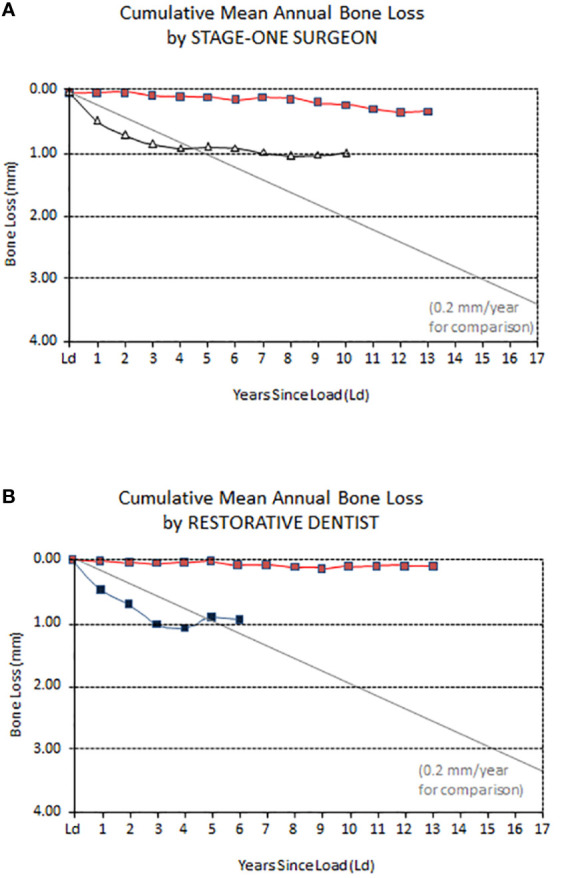
**(A) **This figure depicts the performance of one individual surgeon with respect to the cumulative, average, annual loss in marginal bone that was associated to this clinician (squares) whereas the triangles depict the average annual performance of another oral surgeon who saw much greater accumulated bone loss than his peer, despite them using the same implant type in similar patients. Both these surgeons were active at the University of Toronto, Canada. **(B)** The squares in this figure represent the cumulative, annual loss in marginal bone associated to two restorative dentists active at the University of Toronto, Canada some 20 years ago. The bone loss curves were constructed so that they started from zero levels by the investigator S Ross Bryant. Figures **(A, B)** indicate that if a given patient had the poor luck to be treated by the least good surgeon and followed up by the least good prosthodontist, this meant an average accumulated loss of marginal bone of 2 millimeters at about 3-4 years after implant treatment. These curves support the notion that marginal bone loss around oral implants need not always have a septic background.  Created using data from S R Bryant, Ph D thesis, University of Toronto Canada.

Immune homeostasis of alveolar bone can be directly affected by microorganisms as noted above, However, new evidence shows that mechanical stimulation could promote the conversion of myeloid-derived monocytes into an activated state, suggesting that occlusal force could drive the immune microenvironment difference between alveolar and long bone. In fact, within the complex immune sensing microenvironment of the alveolar bone (44), alveolar macrophages are critical during the early stages of osseointegration (45). Therefore, more recent research has pointed attention to a largely aseptic reason for MBL. For example, high levels of oxidants are produced during chronic hypoxia and inflammation leading to bone loss. This leads to tissues or bone becoming hypoxic by losing their vasculature when exposed to overpressure. Conversely, when insufficient pressure is exerted on bone due to lack of mechanical activity, oxidant production also increases (46). As described above, bone cells such as osteoblasts and osteoclasts have been identified as not only bone building and bone degrading cells but also as a functioning part of the immune system (47, 48). The skeletal system and immune- and inflammatory systems seem independent of one another but, in fact, are inseparable and closely related (49). The aseptic mechanism of MBL may simply be viewed as the immune system stimulating macrophage and osteoclastic function more than osteoblastic activity which inevitably will lead to bone resorption. Osteoblasts and osteoclasts have long been known to be functionally coupled to one another (50). More recently, the data is overwhelming that both these cells act as part of the broader immune system (51). Other factors known to cause MBL such as unsuitable oral implant designs (25), clinical handling (activities of individual surgeons/restorative dentists (**Figures 3A, B**) (52) or, pharmaceutical treatments (53) are in all probably aseptic in nature. Other aseptic causes of MBL may be disuse atrophy and, possibly, resorption due to old age of the implant host. Most certainly, there are many cases when it is uncertain whether the origin of MBL is septic or aseptic or their combination.

### 4.3 Ligature model in question

A great number of “ligature studies” have been published, allegedly serving as the experimental approach to prove the bacterial origin of MBL (54). However, when ligatures were placed around implants in tibial sites, not known to harbor any bacteria, some interesting findings were reported. Firstly, there was a clearly enhanced immunological response to implants with ligatures compared to control implants without ligatures. Secondly, despite the apparent absence of bacteria, MBL was observed anyhow around implants with ligatures, but not around controls without ligatures (55). These findings from long bones of animals indicate a general relevance with respect to the noticed increase of immune reactions to ligatures, a new observation that in all probability would be present as a primary reaction also in maxillofacial bone. However, in the latter site there are numerous bacteria too and, particularly if the immune system is repeatedly provoked by the placement of new ligatures at two week intervals (54) as is commonly done, a rejection phenomenon will occur with due lowering of the bacterial defense leading to implant failure. Researchers in these cases, may not have known about nor appreciated the immune reactions to implants and ligatures/ligature placement in the past, hence they have generally not been concerned with this strong provocation of the immune system. In light of our new knowledge however, ligature studies appear to be excellent at provoking an immune dis-equilibrium and initial aseptic bone resorption. When the ligature-provoked immune system switches over from a shield-off reaction to rejection, the contribution of the ligature trauma and ligature accumulated bacteria to the observed bone resorption is unknown but appears to be similar to what is observed around failing clinical implants.

## 5 Stages of osseointegration failure

**Figure 4:**
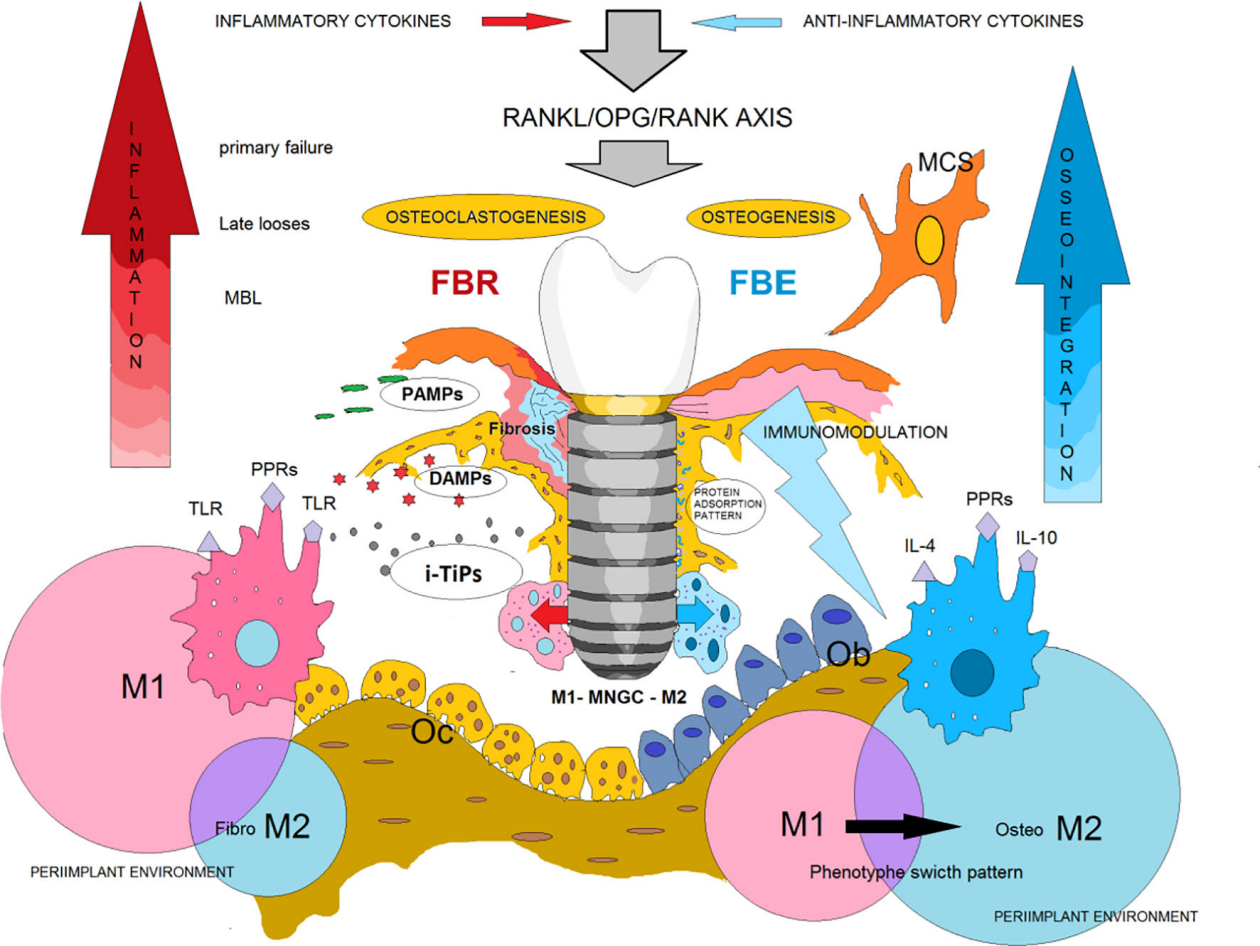
Implant-Osteoimmune interaction. Osseointegration is a condition of continuous and dynamic implant-osteoimmune interaction. If the implant surface evokes an initial and long-term immunomodulation, interfacial bone is formed to shield off the implant from the tissues (FBE). In addition, the M2 anti-inflammatory environment would induce adequate defense reactions to handle transient septic and aseptic threats (PAMPs, DAMPs, Implant-derived Titanium particles (i-TiPs) ), which is clinically reflected with 10 year failure rates varying between 0-4%. However, if it is continuous and of considerable size, the provocation and the consequent M1 inflammatory environment can generate Inflammatory cytokines that alters the expression of RANK/RANKL axis, counteracting the ability of implant surface osteoimmunomodulation, then a partial, progressive or total FBR can occur. Modified from Zetao Cheng, et al (ref.^20^).

During the last years, the concept of osteoimmunology has been highlighted, and osseointegration seems to be a foreign body reaction (FBR) equilibrium whose mechanism depends on a complex cellular heterogeneity and dynamic changes within the implant-mediated osteoimmune microenvironment. This was demonstrated in a recent study that mapped the general osteoimmune microenvironment around the bone implant through single cell RNA sequencing, scRNA-seq (56). Under this biological context, it has been suggested that primary (early) failure, MBL, and periimplantitis (late loss/failure) are clinical terms that, respectively, describe a picture of early, transitory or late breakdown of osseointegration (57). In recent years, thanks to the better knowledge of immunologically caused tissue responses, it is understood that these so-called “biological complications” could be related, and it is possible that they represent different manifestations of the same condition, that is, a local peri-implant imbalance of the innate immune system, either site specific (MBL) or involving the circumference of the shield-off bone (10). Therefore, a possible mechanism may be that a balanced plasticity in peri-implant macrophages could be related to a long-term FBE. On the contrary, an increase in the M1/M2 ratio (imbalance) could be behind peri-implant bone loss, likely a clinical manifestation of an incipient or ongoing FBR (**Figure 4**).

### 5.1 Primary or early failure

Primary failure is the clinical scenario where osseointegration is never achieved. The frequency of such failures is low, in the range of 0–2% in most clinical reports (57). Clinically, this corresponds to oral implants that are found to be mobile at the abutment connection, and already before the placement of the definitive prosthesis and in the absence of other pathological signs. The major histologic findings show that such implants are surrounded by a connective tissue capsule. Also, in some cases, an epithelial down growth is observed with epithelial cells attached to the implant surface *via* hemidesmosomes (58).

In the field of bone biomaterials, it is known that a prolonged M1 polarization phase leads to increased fibrosis-enhancing cytokine release pattern by M2 macrophages, resulting in the formation of a fibrocapsule (20). In fact, in an animal model of osseointegration, a prolonged M1 polarization phase with high M2 phenotypic activity was demonstrated around copper when compared to titanium, and the formation of a fibrocapsule around copper was observed (36). It is known that when M2 macrophages take an important pro-fibrotic role it is because the lesion is persistent in that environment. M2 cell populations are known to be able to secrete large amounts of pro-fibrotic factors such as TGF-β and Galactin-3 (59). Interestingly, M2 macrophages can also induce the epithelial-to-mesenchymal transition (EMT) through TGF-β (60). EMT, in turn would play a role in the development of fibrosis, as the matrix-producing myofibroblasts arise from cells of the epithelial lineage in response to injury (60). In this sense, a link has been proposed between EMT, fibrosis and foreign body response (61). In addition, M1/M2 imbalance on copper, could be related to a non-enzymatic oxidation catalyzed by Cu2+ and the generation of host-derived oxidation-specific epitopes, which represent danger associated molecular patterns, DAMPs, whose major mechanism of recognition is *via* pattern recognition receptors (PRRs) primarily expressed on macrophages (62). Therefore, a similar mechanism could hypothetically be related to primary failures.

Indeed, several DAMPs and their accompanying PRRs have been associated with the activation of inflammatory responses, wound healing and biomaterial implantation, especially in non-infectious environments. Recently it was demonstrated that the inhibition of HMGB1 (prototypic DAMP) or receptor RAGE impair osseointegration, resulting in a foreign body reaction with persistence of M1 macrophages, necrotic bone, and the presence of MNGCs (63). In turn, a prolonged M1 polarization phase may be dependent on cytosolic multiprotein oligomers of the innate immune system responsible for the activation of inflammatory (inflammasome) activation, creating a pro-inflammatory environment susceptible to bone resorption (64). Specifically, the NLRP3 inflammasome senses a variety of signals referred to as DAMPs, including those triggered by degradation products of the extracellular matrix. Thus, the bone DAMP/NLRP3 inflammasome axis has been proposed as a novel mechanism that sustains bone resorption, mainly at conditions of low-grade inflammation (65). In addition, low-grade inflammation decreases access to oxygen and nutrients in affected tissues. Hypoxia could then lead to tissue necrosis, thereby increasing the local immunogenicity *via* the generation of DAMPs (66). On the other hand, the epithelial downgrowth observed on implant failures may therefore be related to the role of M1/M2 macrophage balance in EMT/MET (mesenchymal epithelial transition) plasticity (67).

### 5.2 Late implant failure

Late losses (after prosthesis placement) can sometimes be attributed to overload and/or secondary corrosion, or to a combination of these. In advanced failure cases, there is an excessive loss of marginal bone, implant mobility and interestingly, the presence of a stratified connective tissue (capsule). Further epithelial downgrowth migration is observed (58, 68). Recently, it has been shown that this could possibly be due to the repolarization of both M1 to M2 and vice versa, and that the macrophage phenotypes are defined by the current cellular microenvironment (24). Moreover, MNGCs present at implant interfaces have also the potential to shift between pro-inflammatory M1-MNGCs (often previously referred to as FBGCs) and wound-healing M2-MNGCs polarization states, whose precursor cells are thought to be derived from osteomacs (69, 70). It is important to note that M1-MNGCs may express a different repertoire or concentration of inflammatory factors (cytokines and chemokines), which are also time-dependent if M1-MNGCs switch towards an anti-inflammatory phenotype. Therefore, the FBR could differ between different biomaterials (71). In fact, the results of FBR, such as chronic inflammation, excessive granulation, collagen fiber deposition, and fibrous tissue formation, are related to the persistence of a microenvironment with upregulation of genes related to inflammation (IL- 1) and the ability of the biomaterial to continue serving as an immunomodulator (72). These are critical findings, because macrophages and other cells of the innate immune system respond to a myriad of signals emanating from their local environments, including signals resulting from the interaction between prosthetic byproducts and periprosthetic cells (66).

DAMPs can be products of necrotic or stressed cells as a result of long-term ischemia and/or toxic effects of prosthetic debris. For this reason, several studies have examined the role of DAMPs in periprosthetic osteolysis (PPOL) (66), as there are several potential sources of ions and particles in implant dentistry (73). Moreover, presence of organic and inorganic contaminants onto some surfaces (74) and the potential exposure of less stable elements such as vanadium and aluminum after surface modification procedures, can also trigger an inflammatory response (75). Regarding Ti ions and particles, it is known that both can coexist in the peri-implant environment. A recent study showed that metal particles embedded in an experimental rat mandible defect triggered chronic inflammation with a foreign body granulomatous reaction characterized by the presence of histiocytes and MNGCs, i.e, Ti metal particles induced a chronic inflammatory cell infiltrate associated with a foreign body reaction (76). Interestingly, new evidence suggests a spatiotemporal distribution of macrophages in the FBR, therefore, a microenvironment may exist or be created within and around the biomaterial and that different macrophage phenotypes are associated with these different spaces (77).

Human macrophages develop a specific response to Ti particles. Upon contact, M1 exhibits increased production of pro-inflammatory cytokines, chemokines and growth factors, but a decreased phagocytic activity, while M2 macrophages have been suggested to mediate particle uptake (78). This could be related to the absence of MNGC or frustrated phagocytosis in the vicinity of titanium particles in granulation tissue harvested from peri-implantitis cases, as shown in a recent article, even though there was a significantly higher expression of CD68 (79). For example, it has been shown that proinflammatory M1 macrophages predominate in soft tissue biopsies from peri-implantitis sites over M2 macrophages (80, 81). As indicated in a recent paper (4), qPCR-techniques were used to verify such immune responses. However, measurable foreign body reactions are a shortlived phenomena and M1-MNGCs may not be possible to study in chronic specimens as done in a recent paper (79). In normal foreign body reactions, M1-MNGCs and associated granulomatous tissue are formed at approximately 4 days after implantation, increase up to about 14 days, but subsequently gradually disappear (82) to be replaced with other immune derived reactions such as machrophage responses. The M1 polarization observed in peri-implantitis lesions also suggests a robust response by the immune system against local factors; and thus, more tissue destruction (81). We should keep in mind that reactive oxygen species (ROS) always dissolve some Ti-oxide during an inflammatory phase. One plausible interpretation is therefore that later dissolved material is “shielded off” due to local immune activation, very similar to the later shield off of macroscopic implants. Inflamed tissues maintain a persistent low level of inflammation and thereby enhance over time the dissolved material that precipitates to particles and necessitate a response, a “shield off” process, or alternatively, a low response due to immunecompromized tissues in the vicinity of implants.

## 6 Marginal bone loss from different perspectives

At present, it is thought that an increase or decrease in bone response is related to implant mechanical stability and the initial response modulated by the immune system (40). In fact, macrophage ablation impairs woven bone formation around oral implants (45), and the impact on the immune response by Vitamin D deficiency has been related to low early implant healing (83). Furthermore, it is known that some intraoral sites support osseointegration better than others. In this sense, studies revealed a strong positive correlation between bone remodeling rate, mitotic activity, and osteotomy site healing and high endogenous Wnt signaling (84). Also, findings suggest a role for an autocrine Wnt signaling in macrophages during the immune response to implanted biomaterials (85).

Histologically, osseointegrated oral implants show a heterogeneous interface with variable degrees of mineralized bone-implant contact (BIC) (86). Therefore, in some cases, there could be a mechanically weak bone-to-implant interface (87). This is clinically relevant since functional loading and mechanical strain are the main causes for bone remodeling. Osteocytes are known to translate signals related to mechanical strain into biochemical signals and largely regulate the osteoblast–osteoclast axis. As a result, bone remodeling may change the peri-implant crestal bone contours (87). In turn, the macrophage-osteoclast axis is involved in regulating the balance of bone remodeling and resorption that is essential for the maintenance of normal bone morphology (88). On the other hand, the rate of new bone formation depends also on proteins secreted by macrophages that regulate undifferentiated mesenchymal cells to transform to bone-forming osteoblasts (89).

The activation of inflammatory processes is followed by physiological bone repair mechanisms. However, there could be typical individual mediator-related signaling patterns of inflammatory cytokines. In this sense, a unique bone remodeling situation appears to occur when fatty degenerative tissue is present in the medullary cavity of the jawbone, which could be related to a dysregulated programming in stem cell expansion (90). Recent findings demonstrate that alveolar bone monocytes/macrophages tend to express a high level of oncostatin M (Osm), which promotes osteogenic differentiation and inhibits adipogenic differentiation of MSCs (44). Therefore, if there is a weak bone-to-implant interface, associated personalized signal patterns, continuous stress signals and immunogenicity of the elements present, there is a risk that initially transitory and site specific peri-implant bone loss may progress to a more damaging and vicious stage (91). Such a mechanism may be especially evident at the marginal bone area.

### 6.1 Macrophage polarization and the osteoimmunological mechanisms behind marginal bone loss as a condition but not as a disease

Macrophages are highly plastic cells that rapidly respond to their microenvironment by adopting different phenotypes with important roles in regulating the healing response to biomaterials. The prolonged presence of inflammatory M1 macrophages can exacerbate tissue damage and prevent biomaterial integration. In contrast, the immune response favorable to healing by M2 macrophages precedes osteoinduction. In recent years, an increasing number of studies have investigated the response of M2 macrophages to biomaterials. In fact, the interaction between M1 and M2 dominated microenvironments and the temporal modulation of the M1 to M2 transition provide an interesting line of investigation to search for new therapeutic approaches focused on the immune system to improve osseointegration. Such studies include modification of implant surface properties, ionic-treated implant surfaces with LiCl or Mg, use of polarizing cytokines such IL-4 and mechanical stimuli to promote the innate immunomodulatory capacities of BMMSCs (91).

Peri-implant tissues may thus be considered as an immunologically active microenvironment with immunological sentinels present such as macrophages modulated by neutrophils, dendritic cells, T-cells, B-cells and MNGCs being able to activate and direct an immune-mediated and controlled inflammatory response (91). Furthermore, it is known that prolonged inflammation plays a critical role in bone resorption, because pro-inflammatory cytokines (such as IL-17A) (92) alter negatively the RANK/RANKL axis balance (93). In this sense, proinflammatory M1 macrophage polarization can be induced by implant/wear debris, damage associated molecular patterns (DAMPs), and pathogen associated molecular patterns (PAMPs), resulting in the production of high levels of pro-inflammatory cytokines (e.g. TNF-a, IL-1b, IL-6) through NF-kB activation. In addition to secreting cytokines, M1 macrophages show potential to differentiate into osteoclasts, and may serve as an osteoclast reservoir. Conversely, M2 activation is often characterized by the expression of anti-inflammatory cytokines (e.g. IL-4, TGF-b and IL-10) and antigen presentation ability, suppress osteoclastic activity and promoted osteogenesis through the inhibition of NF-κB signaling pathway (94, 95). Although the mechanism underlying the observed plasticity in macrophages is not well understood, It is thought that macrophage polarization represents a “fluid state”. In this regard, polarization reversibility is a target of therapeutic interest, especially when the M1/M2 imbalance may compromise the immune response (96). In a recent study, researchers analyzed the subpopulations of M1 (CD68 and iNOS) and M2 (CD68 and CD206) macrophage polarization through Immunofluorescence staining, noting a statistically significant increase in population of macrophage M1 phenotype from peri-implantitis samples compared to periodontal disease samples. In the same line, an immunohistochemical analysis showed a significantly higher expression of M1 (CD80) inflammatory phenotype at advanced peri-implantitis sites (80, 81). These studies correlate the increase of the M1/M2 ratio with a high response of the immune system against local signals in the cases of peri-implant lesions, which could possibly play a critical role in the underlying pathogenesis of peri-implant bone loss (80, 81).

### 6.2 Implant-abutment site and marginal bone loss

The connections of various implant components to the top of the implant and their emergence from the body’s hard and soft tissues have implications for tissue attachment and turnover. Generally, components are placed, removed and replaced on multiple occasions including closure screws, healing caps, temporary abutments, final abutments and temporary and final restorations. These component placement and removal procedures not only prevent stable soft tissue attachment onto the implant component but also provide an avenue for fretting and galvanic corrosion, and bacterial access to interfaces including the interface at the top of the implant. Many studies have documented bacterial contamination of these interfaces regardless of whether the connections are internal or external to the implant (97). These contaminated interfaces therefore provide an ecological niche for bacterial colonization and their products such that the host response is unable to eliminate or mitigate the bacterial challenge. As such, the host must provide an immunological response adjacent to the interface. Clinicians generally place the top of a bone level or “submerged” implant at or slightly below the crest of the bone meaning that a bacterially contaminated interface, and consequently, a host inflammatory reaction is located directly at the marginal bone level.

Broggini et al. (98) documented that a peak of inflammatory cells was located approximately 0.50 mm coronal to the interface in tissues adjacent to the implant. This inflammation consisted primarily of neutrophilic polymorphonuclear leukocytes indicative of a persistent acute inflammatory reaction at the marginal bone level. Mononuclear cells were evenly distributed along the implant surface, and this inflammation was associated with bone loss. Interestingly, the absence of an interface at the bone level (using a tissue level or “non-submerged” implant) resulted in only sparse cells and no peak of inflammation at the marginal bone level and minimal bone loss (98). The peri-implant cellular infiltrate immediately coronal to the implant-abutment interface decreased gradually and progressively in the soft tissues toward either bone or gingival epithelium. This study provided histomorphometric data that a unique pattern of inflammatory infiltrate develops adjacent to implant interfaces with associated bone loss. The differential pattern of peri-implant neutrophil accumulation suggests that the bacterial accumulation at the interface results in a chemotactic stimulus that both initiates and sustains the recruitment of inflammatory cells. Such activation of the host defense system (such as cytokines, complement, and antibodies) can result in a gradient of inflammatory cells perpetuating an acute inflammatory process which is exacerbated by an inability to access the interface for oral hygiene (98). This study, in addition to documenting the intense inflammatory process, also demonstrated significantly greater bone loss around implants with an interface at the marginal bone level compared to implants without such an interface (98). It was hypothesized that the interface at the marginal bone level leads to microbial leakage, colonization and a persistent bacterial presence. The chemotactic signaling promotes a sustained neutrophil accumulation and, in parallel, mononuclear cells are recruited to the surface. The combined and sustained activation of inflammatory cells can then promote osteoclast formation and activation resulting in marginal bone loss.

Another study compared the distribution and density of inflammatory cells surrounding implants with an implant-abutment interface placed supracrestally, at the crest or, subcrestally and correlated that with bone loss (99). This study revealed that, in spite of location, all implant interfaces had a similar pattern of peri-implant inflammation. That pattern consisted of polymorphonuclear leukocytes concentrated at or immediately coronal to the interface. Interestingly, peri-implant neutrophil accumulation increased progressively as the interface depth increased and marginal bone loss was significantly correlated with inflammatory cell accumulation, i.e. the deeper the interface, the greater the magnitude of peri-implant inflammation (99). In contrast, mononuclear cells were relatively uniformly located along the entire surface of the implants. Furthermore, there was significantly greater bone loss associated with subcrestal implants compared to implants placed at the crest or supracrestally. These findings reveal that the implant-abutment interface defines the degree of inflammatory cell accumulation and its location in the tissues and, suggests that the inflammatory cells contribute directly or indirectly to the extent of marginal bone loss (99).

The study above identified a highly significant relationship between the degree of peri-implant inflammation and the magnitude of marginal bone loss. A number of previous studies have also demonstrated a spatial relationship between inflammation and bone loss supporting the observed association between contaminated implant-abutment interfaces, inflammatory cell infiltrate accumulation and marginal bone loss (100, 101). In the late 1970’s, Waerhaug (100) described in periodontal disease an “extended arm” of inflammation while Garant (101) described an “effective radius off action” of inflammation to bone loss. More recently, Graves and Cochran (102) described such a relationship as an “inflammatory front” where an increase in the host inflammatory response resulted in an increase in bone loss. This cause-and-effect relationship was demonstrated with inhibitors to the pro-inflammatory molecules IL-1 and TNF-alfa (103). This spatial relationship between inflammation and the immune system and bone has resulted in an area of science referred to as “osteoimmunology” as noted above and involves the science related to osteoclast development (104, 105). Taken together, these studies demonstrate that the location of an implant-abutment interface can be an important determinant of marginal bone loss as has been noted when evaluating marginal bone loss for implant success (106) where up to a mean of 1.5 mm of marginal bone loss was allowed for in the first year after implant placement.

In summary, bacterial-induced inflammation and corrosion may together with other factors contribute to MBL by jointly affecting peri-implant bone rather than as isolated factors. Secondary corrosion is a late implant response that may, in clinical cases which have previously resulted in some MBL, facilitate a transitional shift in the immune system from being a sentinel of implant shield off, to implant rejection, even if this is not an inevitable outcome of secondary corrosion (107) that will be discussed in greater detail under next heading.

## 7 Peri-implant phenomena involved in osteoimmune regulation

### 7.1 Implant passivation layer

**Figure 5:**
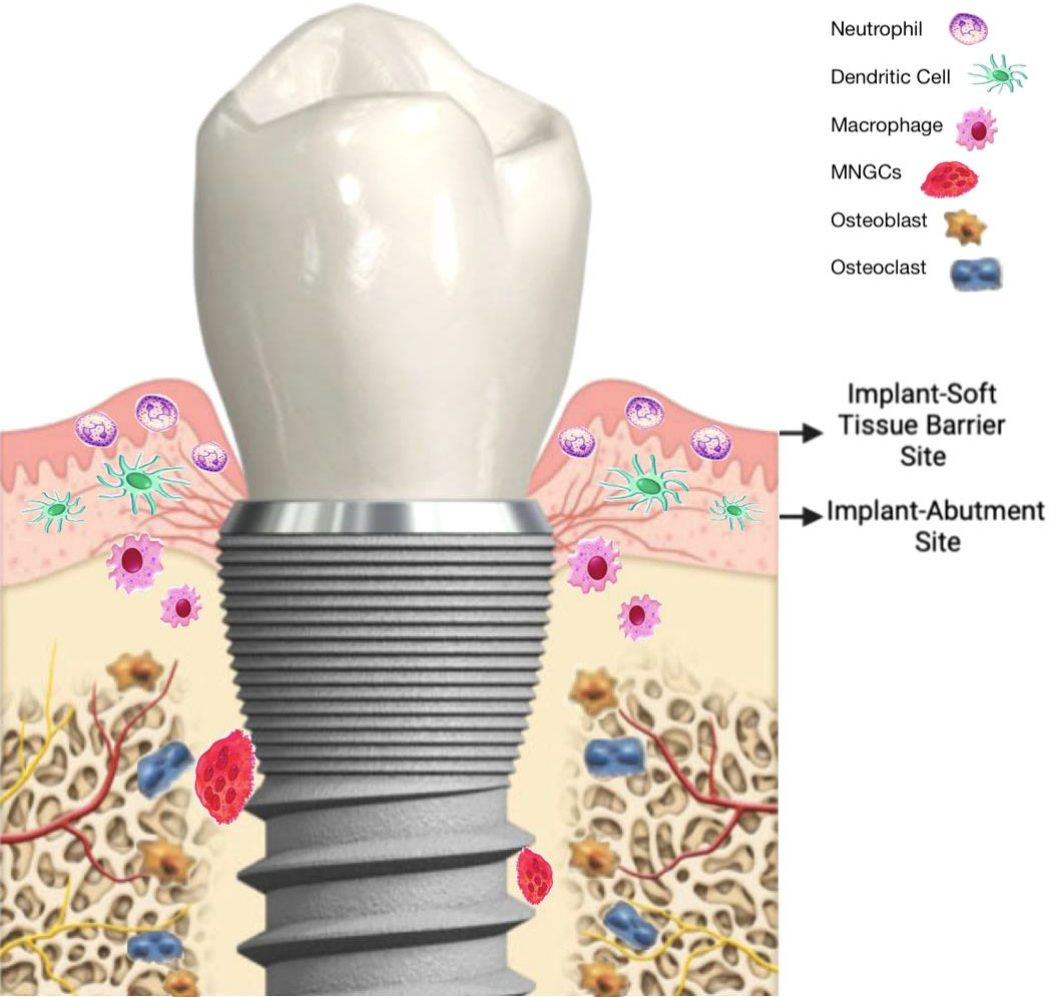
Two critical sites involved in marginal bone loss exist at the coronal aspect of the implant where it emerges through the bone and soft tissues.

The coronal portion of the implant exists in a spatially singular situation where it interacts directly and simultaneously with the oral microenvironment (**Figure 5**), the peri-implant soft tissue barrier. As discussed previously in this article, no biomaterial is fully bioinert. However, select non-toxic biomaterials such as titanium can achieve a homeostatic state within the peri-implant tissues enabling a long-term functional stability (108). This state is dynamic and contingent upon the biomaterial’s capacity to reach an electrochemical equilibrium, while present in biological fluids. For titanium biomedical implants, the success of primary osseointegration is dependent upon the establishment of a surface “passivation” layer (109, 110). The chemical composition of this layer is distinct from that of the underlying metal, being mainly (>98%) composed of titanium dioxide, TiO_2_. The passivation layer is formed rapidly but not instantly on titanium surfaces under atmospheric conditions and protects from further passive oxidation of the implant. Therefore, it contributes to the long-term stability of the implant within the tissues without further corrosion. The establishment and development of the passivation layer is also dynamic and the electrochemical changes that occur due to insertion of the implant in an osteotomy within the bone result in electrochemical changes that move hand in hand with the process of osseointegration. During successful osseointegration the passivation layer thickness maximizes, while a direct bone-to-implant contact is established and maintained (110). Importantly, osseointegration is achieved between the titanium passivation layer and host bone cells, and not between the underlying metal and host tissues (111). In fact, no published data has ever shown cellular attachment on titanium surfaces without protective passivation layers.

In essence these electrochemical changes that occur at the titanium surface represent a controlled primary corrosion of the metal under the definition of passivation as the “conversion of a refined metal into a more chemically stable form, such as the spontaneous formation of an ultrathin film of corrosion products, known as a passive film, on the metal’s surface that act as a barrier to further oxidation” (112). As mentioned previously (see **Figure 2**) immune and bone cell populations respond to these early electrochemical events that occur during implant osseointegration with a specific role being played by alveolar macrophages during the early stages of osseointegration (4, 45). In addition to the direct signaling of the RANKL-OPG pathway that occurs in response to the surgical trauma induced to osteocytes during implant placement, at least two independent *in vivo* animal models have demonstrated that the first two- (rat model) (45) to four-weeks (rabbit model) (4) of the osseointegration phase are dominated by CD68+ macrophages expressing both M1 and M2-related genes, suggestive of a inflammation-driven remodeling. Depletion of macrophages in the rat model led to compromised osteogenesis during early osseointegration, highlighting the central effect that immunity has in regulating the biomaterial-bone interface (45). The important role of implant surface passivation in ensuring an optimal tissue response to the implanted metal is evidenced by the fact that when the implant passivation layer thickens, as in the case of Mg-oxidized implants (113, 114), that increased thickness of the passivation layer provides improved bone anchorage.

### 7.2 Implant-soft tissue barrier with focus on inflammation and primary corrosion

When discussing host immune/inflammatory responses to biomaterials it is important to destigmatize the term “inflammation” because it has traditionally been linked to the host defense process against harmful microorganisms. However, it is now well established that inflammatory responses are part of host physiology and are necessary processes to regulate tissue and organ function, wound healing and cell death. Inflammation is therefore critical to eubiosis (115) and not necessarily results in tissue destruction (116). Inflammatory responses only become implicated in the pathophysiology of diseases when they become deregulated, non-resolving and as a result become chronic. In the context of implant biomaterial-host equilibrium, successful osseointegration is characterized by a controlled immune/inflammatory response that is critical to peri-implant wound healing and, in most cases, resolves timely to allow chronic immune surveillance to aid in maintaining tissues homeostasis. Nonetheless, if the tissue environment is not conducive to the electrochemical stability of the titanium passivation layer, destructive corrosion can occur leading to titanium dissolution from the implant surfaces (107, 108). Wennerberg et al. (117) addressed the extent of primary corrosion during the osseointegration of titanium implants with various surface modifications by artificial material aging in solution for 1-month at atmospheric conditions. None of the implant surfaces exhibited dissolution of titanium from the surface during the experiment in buffered saline suggesting that an electrochemical equilibrium is rapidly established and sustained under favorable conditions, which resemble healthy tissue, i.e. oxygen availability, neutral pH=7.3 (117). However, when the same surfaces were placed in strongly acidic lactate solution (pH=2.3) and aged for 1 month up to 250ng of dissolved titanium were identified in solution (117). Therefore, aggressive electrochemical conditions, such as a strongly acidic environment or chemically reductive conditions, may lead to electrochemical instability of the passivation layer and titanium release *in vitro* even in the absence of bacterial and frictional challenges (107). Vascular interruption as a result of surgical trauma in the case of implant placement is another example of a micro environmental factor that may contribute to electrochemical instability. In corroboration, a separate study (118) showed that the corrosion resistance of titanium is diminished under inflammatory conditions that included oxidative attack by reactive oxygen species (119), acidic environment (pH~3) and reduced oxygen availability (anaerobic conditions in peri-implant pockets) (118). Among these environmental factors, lack of oxygen achieved by de-aeration was the strongest determinant of diminished electrochemical impedance (118). Although these environmental challenges have been described from a biomaterials viewpoint, it is clear that they are bidirectional and affect the host tissues as well. When the electrochemical equilibrium on the titanium passivated surface is displaced, more titanium ions are generated and dissolved in tissue fluids. It has been suggested that these titanium ions rapidly aggregate in protein-rich fluids forming highly biologically active titanium microparticles (119, 120).

### 7.3 Implant passivation layer and secondary corrosion

When the chronic electrochemical oxidation of titanium leads to gradual destruction of the passivation layer, the effects of corrosion are not limited to the biomaterial but also affect osteoimmune regulation of osseointegration. This has been evidenced by two recent studies (108, 121) from independent research groups showing that abrasive dental treatments, such as ultrasonic instrumentation with steel instruments used to clean the implants surface, leads to destructive corrosion. This can be regarded as secondary corrosion when compared to the primary oxidation, i.e. corrosion, which occurs during healing of implants and has a protective effect in most cases *via* the formation of the passivation layer. In the case of secondary corrosion, the resulting damage to the passivation layer results in accelerated titanium release from the implant surface to the tissues with detrimental effects locally and deregulation of the osteoimmune axis (107, 108). It was long thought that the scratch exposed metal would, however, be re-oxidized in water/air within tens of milliseconds to seconds (122) as the re-passivation of titanium in water or air is an undoubtable scientific fact. Nonetheless, it is not translational to the dental implant clinical reality. Earlier studies were conducted in atmospheric conditions or in water but neither of these conditions represent the microenvironment of the peri-implant pocket. As a result, the fallacy that clinicians can damage the implant surfaces to “clean” them from bacterial biofilm was developed under the assumption that the titanium passivation layer will re-passivate after abrasion within milliseconds (108). Conversely, Berbel et al. (108) showed that when replicating anaerobic inflammatory conditions that exist in the peri-implant pocket to repeat these experiments, scratching of the passivation layer for cleaning resulted in long-term reduction in corrosion resistance. These changes led to secondary corrosion appearing as microgranular corrosion on the titanium surfaces (108, 118). In a subsequent paper it was further shown that these abrasions of the passivation layer led to vastly accelerated titanium release to the environment in simulated body fluid during titanium aging. As such, it is imperative to highlight that the notion that titanium will rapidly re-passivate does not stand true under clinical conditions.

These findings have important clinical ramifications to avoid initiation or perpetuation of peri-implantitis due to iatrogenic reasons, such as preventive abrasion of implants with steel instruments to remove bacteria. Importantly, the released implant-derived Titanium Particles (i-TiPs) cause fibroblast cell death and activate macrophages towards an M1 phenotype (108, 121). Importantly, the persistent effect of i-TiPs activates inflammasomes in immune cells that lead to IL-1β release through activation of the complement system (4, 123, 124). As discussed above, IL-1β is a major osteoclast activating factor and provides a means of communication from immune and tissue resident cells to the local bone eliciting osteoclastic differentiation with destructive downstream effects. Therefore, the biological plausibility exists for regarding the electrochemical instability of the titanium surface occurring either through tribocorrosion (i.e. surface transformations resulting from the interaction of mechanical loading and chemical/electrochemical reactions), local chemical attack (ROS or Fluorides) or damage by dental implant instruments as a potential cause of marginal bone loss within the implant-soft tissue barrier Interface.

## 8 Synergistic activation of pro-inflammatory pathways

Macrophages and other cells of the innate immune system respond to a large number of signals emanating from their local environment; therefore, the inflammatory potential can be multiplied due to the synergistic activation of pro-inflammatory pathways. As described above, proinflammatory M1 macrophage polarization can be induced by implant/wear debris, DAMPs, and PAMPs (95).

It appears that titanium particles do not tend to be encapsulated in the tissues around dental implants, but instead migrate through peri-implant tissues causing immune reactions, with smaller particles tending to produce greater toxicity and enhanced pro-inflammatory response (125). In relation to this, it is known that particles of a diameter smaller than 1 µm, or nanoparticles, generate the most biological toxicity and can induce cellular mutations. In a recent study, it was shown for the first time that Titanium nanoparticles (TiNPs) affect the transcriptional program in human macrophages (GDF-15 over-production and strong suppression of stabilin-1), which could interfere with the long-term integration of the implant through the imbalance between inflammation and healing processes (126).

While the molecular mechanism of DNA damage induced by TiO_2_ NPs is unknown, it is suggested that exposure to TiO_2_ NPs causes aberrant DNA methylation levels that can lead to unusual gene expression, altering epigenetic integrity (127).

It is observed that the macrophage reactivity upon activation by wear particles is driven by cell membrane contacts through surface receptors, such as CD14 and TLRs (128), or through the phagocytosis of wear debris and the stimulation of the NALP3 inflammasome(NLRP3, Cryopyrin) (129). In bone and its surrounding tissues this results in an influx of immune cells, osteoclasts and other cells. The resulting pro-inflammatory environment leads to increased bone destruction and suppressed bone formation (130).

**Figure 6:**
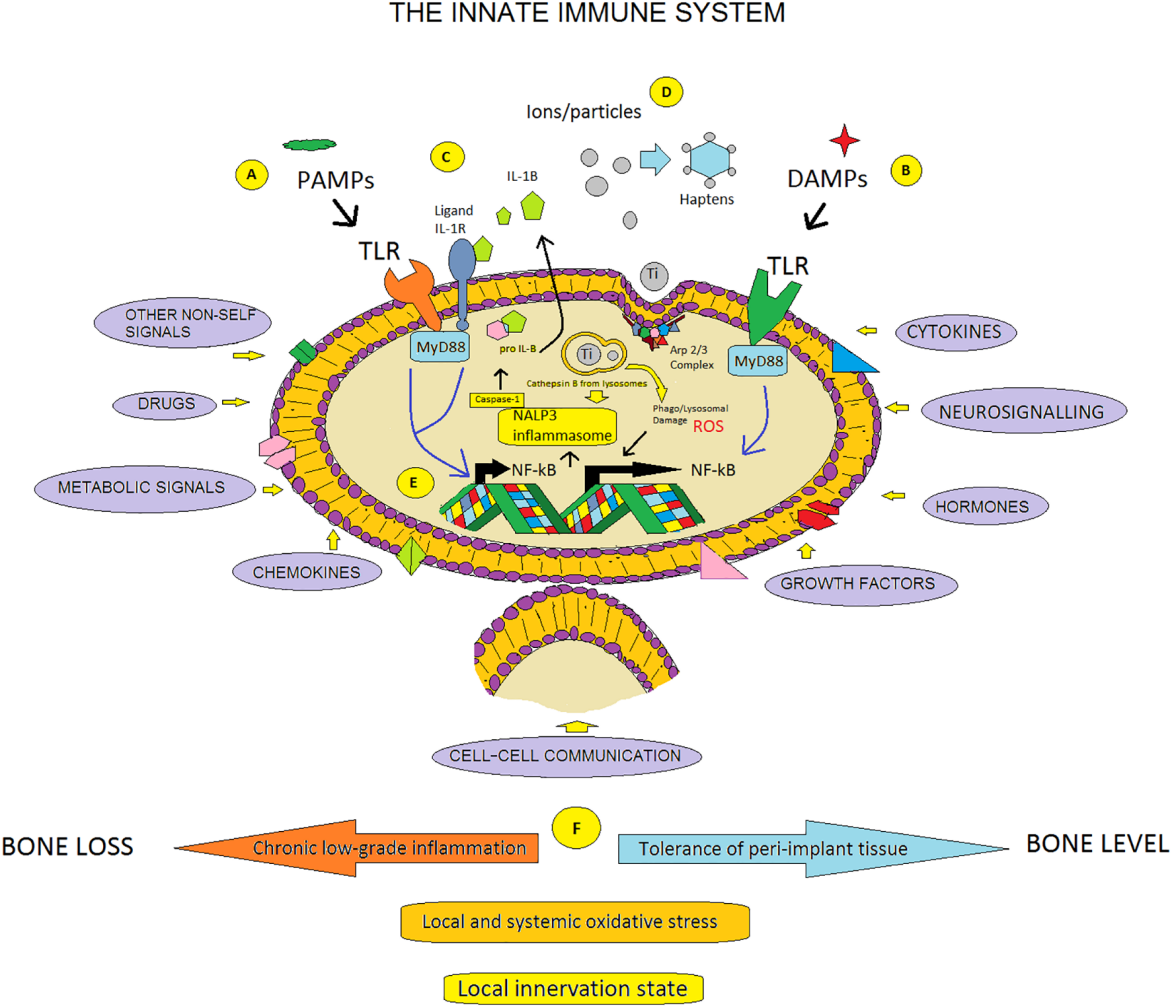
Common molecular pathways and environmental signals. **(A, B)** Toll-like receptors (TLRs) and other types of pattern recognition receptors recognize PAMPs and DAMPs and trigger inflammation via the activation of the transcription factor NF-Kb. Signaling pathway that requires the adaptor molecule MyD88. **(C)**  In addition, inflammation in response to necrotic cells is mostly mediated by IL-1 receptor (IL-1R), which leads to NF-kB activation.  **(D)** On the other hand, titanium particles can induce acute inflammation due to activation of the NALP3 inflammasome, which leads to increased IL-1  secretion and IL-1-associated signaling. Process mediated by protein complexes such as the Arp 2/3 complex. Also, titanium ions can bind to proteins, such as albumin or transferrin, creating a bioavailable metalloprotein that could serve as an antigen in immunological reactions. **(E)** Activation of NF-κB , the master inflammatory transcription factor. **(F)** Macrophages and other cells of the innate immune system respond to a large number of signals emanating from their local environment, therefore, the inflammatory potential can be multiplied due to the synergistic activation of pro-inflammatory pathways.  In this sense, it is known that the crosstalk between the skeletal system and the immune system can lead to osteoclastogenesis, for example, through IL-1. A specific biologic response of either inflammation or tolerance in a particular patient could be related to local and systemic oxidative stress, and other basal states, such as the state of local innervation. All these possible cellular and molecular mechanisms would be constantly counteracted/balanced by both the long-term immunomodulatory capacity of the implant and the dynamic osteo immune environment. (Modified from Goodman SB, et al. ref. 66).

It is not known in detail how these molecular and cellular interactions translate into a specific biologic response of either inflammation or tolerance in a particular patient (66). However, the osteo-immune response could be conditioned not only by local and systemic oxidative stress but also by the local innervation state (**Figure 6**). In support of the latter, recent *in vivo* experiments using Ti-implants in rat femur indicated strongly that neural regulation of bone directly modulates its formation and, as a consequence, osseointegration (131). The significance of this finding is not currently understood, but almost certainly there exist tight connections to the immune/inflammatory system. It is well known that both the inflammatory reaction and the wound healing process are intimately connected to changes in the redox balance, and even though at low concentrations, oxidative stress exhibits various physiological roles. Upregulation of Reactive Oxygen Species **(**ROS) production and persistence over a long period of time can then prove to be harmful to the host (132). In fact, recent discoveries, have demonstrated a link between oxidative stress and an aberrant innate immune system response in sterile inflammatory diseases (133).

The general presumption that biomaterial implantation allows opportunistic bacteria to flourish by providing a surface for biofilm formation likely is biased. The dysregulated host response opens the opportunity for bacteria to invade immune compromised tissues and hence contribute to the susceptibility of implants to infection (37). In this sense, the beginning of understanding bone loss as a condition is a great paradigm shift that allows osseointegration to be considered from a different point of view. Reincorporating oral implantology to the field of biotechnology where the emergence of omic sciences such as implantogenomics (134), epigenetic effects of nanoparticles (135) and advanced immunomodulation (136) acquire enormous relevance when maintaining implant health in our patients.

## 9 Concluding remarks

Since periodontitis may cause loss of teeth, peri-implantitis was assumed to cause loss of oral implants with increasing time of follow up. Accelerating loss of marginal bone around implants was, therefore, regarded as a disease that logically, as it seemed, would best be treated by a similar type of surgery as periodontitis. One cannot blame the doctor for interpreting the numerous bacteria present in the end stage of bone resorption to be what caused the problem in the beginning, since there was no alternative explanation for this development that was known at the time.

However, today we have identified alternative explanations behind implant threatening bone loss; adverse immune reactions that can be demonstrated to be behind failure of oral as well as orthopedic implants (11, 137). The science of osteoimmunology is relatively new and has been established first in our new millennium and mainly after the initial attempts to couple all marginal bone loss to a bacterial disease. Furthermore, we recognize today that teeth are natural parts of our human bodies whereas implants represent foreign bodies with clearly measurable immune reactions (4). It is to no great surprise that investigators have demonstrated clear differences between periodontitis and peri-implantitis (107, 138). One study compared teeth and implants in the same jaw of patients and found that when teeth lost bone, implant bone level was stable and, conversely, when implants lost bone, teeth bone was stable. In only 3% of cases was there simultaneous bone loss around teeth and implants reported (139). Surgery for what has been seen as threatening marginal bone loss around oral implants have, at best, presented questionable clinical results with a clear tendency of causing more patient problems than non-surgical approaches (43, 140). In addition, implants with a diagnosed state of alleged disease at a mean of 12.5 years after placement (141) were re-investigated 9 years later when it was demonstrated that 91.4% of the allegedly sick implants had seen no further bone loss and 95.3% of the previously as sick declared implants still functioned in the jaw of the patients (142). In another study, a decreased risk for oral implant losses with increasing time was reported (143). Increasing plaque index was found associated with lower levels of MBL (144) and Menini et al. (52) was unable to find any MBL associated with increasing plaque index in an up to 14 year followed up clinical study. There are indeed several reasons for MBL which are most difficult to explain with a primary infection etiology. These situations include MBL associated with the responsible surgeon or prosthodontist (53), MBL associated with intake of pharmaceutical products (145) and at least initial MBL due to accidental presence of cement in the soft tissues. However, the latter example is of dual nature; MBL due to (nano-micron sized) cement particles will immediately stop if the cement is removed, indicative of this bone loss being immune driven since bacterial actions would not disappear instantly. However, if cement is not removed in time, then the immune system may start a rejection phenomenon whereby a secondary infection will ensue. Taken together, the evidence for functioning, osseointegrated implants suffering from an infectious disease is insufficient. The paradigm shift is that we today know that implants are not bio-inert as previously believed (146); instead an immune system activation follows the placement of an oral implant (4). The immune system has two ways of responding to an implant; either to embed it in bone to protect other tissues (bone shield off; osseointegration) or rejection of the foreign body (3). In the great majority of cases there will be an immune system caused shield-off of the implant. Some marginal bone loss can be monitored by the immune system control of the osteoblast/osteoclast combined action (10). A more dangerous development would be if the immune system is overwhelmed by implant threatening attacks; it may then shift over to rejection of the oral implant.

This view does not exclude the role of infection in particular cases. When the implants have a maintained immune-caused shield-off, there appears to be bacterial protection. However, there may be situations when this protection may not be active and then a direct infection with subsequent MBL is a possibility that can be exemplified by broken implant components where parts of the implants are not stable. Further, we cannot exclude situations when the immune system is overwhelmed by bacteria that then may act as a regulator of the osteoimmune system, e.g. if the immune system is compromised in some way and the normal bacterial flora becomes pathogenic. Bacterial presence may be controlled by the immune system, but the bacteria will always be present and do not disappear. Therefore, in the age of osteoimmunology, one must always remember that, under the right circumstances, it would be sufficient with only a few surface located and slime protected bacteria to cause infection and severe tissue problems, e.g. as described *via* the “race for the surface” mechanisms (147).

## 10 Conclusions

1. Osseointegration is needed for oral implant function.
2. Recent advances in osteoimmunology suggest that osseointegration is an osteoimmune defence reaction, more than a simple bone repair process.
3. The bone-anchored implant integration process should in the future be termed“the immunoinflammatory process” instead of only the “inflammatory process”. In this process the innervation development adjacent to implants is also important.
4. Osteoimmunological mechanisms underlie marginal bone loss (MBL) as a condition, not a disease.
5. The immune system is capable of causing MBL through its control over the osteoblast/osteoclast coupled function.
6. As far as is known today, bacteria may affect oral implants secondarily once a rejection reaction by the immune system has been initiated. Local bacterial reactions, not affecting implant stability, may occur adjacent to leakage from the abutment implant connection.
7. Patient related factors such as smoking, consumption of certain pharmaceuticals and genetic disorders as well as surgical and prosthodontic techniques, local microbes, foreign bodies such as small cement particles, primary corrosion and implant fractures can cause MBL monitored by the immune system. Secondary corrosion may later add to these oral implant survival challenges that, taken together, may, lead to a shift in the immune reactions from bone shield-off to rejection of the implant.

## Author contributions

AT; concept/design, author contribution, data analyses/interpretation. TP; critical revision of article, drafting article. AL; author contribution, critical revision of article. CP; author contribution, concept/design, data analysis/interpretation, drafting article. KG; author contribution, data analysis/interpretation, drafting article. CD; author contribution, data analysis/interpretation, drafting article. All authors contributed to the article and approved the submitted version.

## Glossary

**Table:**

BIC	bone-implant contact
BMMSC	bone marrow mesenchymal stem cell
BMU	basic multicellular unit
CD68	cluster of differentiation 68
DAMP	danger associated molecular pattern
EMT	epithelial-to-mesenchymal transition
FBE	foreign body equilibrium
FBGC	foreign body giant cell
FBR	foreign body response
GDF-15	growth/differentiation factor 15, a member of transforming growth factor beta family
HMGB1	High mobility group box 1 protein
IL-4	interleukin 4
iNOS	inducible nitric oxide synthase
i-TiP	implant-derived titanium particles
M1	macrophage phenotype 1, pro-inflammatory
M2	macrophage phenotype 2, pro-regenerative
MBL	marginal bone loss
MET	mesenchymal epithelial transition
MNGC	multinucleated giant cell
MSC	mesenchymal stem cell
NF-κB	nuclear factor-κB (NF-κB), a transcription factor
NLRP3	NLR family pyrin domain containing 3
NP	nanoparticle
OPG	osteoprotegerin
Osm	oncostatin M
PAMP	pathogen associated molecular pattern
PPOL	periprosthetic osteolysis
PRR	pattern recognition receptors
PTH	parathyroid hormone
RAGE	receptor for advanced glycation end products, a pattern recognition receptor
RANK	receptor activator of nuclear factor κ B
RANKL	receptor activator of nuclear factor κ B ligand
ROS	reactive oxygen species
scRNA-seq	single cell RNA sequencing technology
TGF-β	transforming growth factor
TLR	toll like receptor
TNF-a	tumor necrosis factor alfa
Wnt	evolutionarily conserved paracrine or autocrine signaling pathways
